# The Effect of Genetically Modified Food on Infertility Indices: A Systematic Review Study

**DOI:** 10.1155/2020/1424789

**Published:** 2020-08-13

**Authors:** Parisa Keshani, Mohammad Hossein Sharifi, Mohammad Reza Heydari, Hassan Joulaei

**Affiliations:** ^1^Shiraz HIV/AIDS Research Center, Institute of Health, Shiraz University of Medical Sciences, Shiraz, Iran; ^2^Research Center for Traditional Medicine and History of Medicine, Shiraz University of Medical Sciences, Shiraz, Iran; ^3^Health Policy Research Center, Institute of Health, Shiraz University of Medical Sciences, Shiraz, Iran

## Abstract

*Background and Objectives*. With the increase in the growth of genetically modified (GM) crops, concerns as to the adverse effects of GM crops have risen in the community. The present systematic review seeks to assess the GM plants' potential impacts on the sperm parameters, including sperm head, sperm motility, sperm abnormality, and fertility index. *Materials and Methods*. A structured literature search was independently performed by three authors on Scopus, Web of Science, PubMed, and Embase in January 2019. A total of 1467 publications were obtained by the search strategy after eliminating the duplicates. Moreover, the review only included articles written in English language. Other pertinent peer-reviewed publications were chosen (“snowballing”) from the reference lists in the selected publications. To assess the GM crop effects on infertility, experimental studies designed with the control group were selected. On the basis of abstract screening and full-text reviewing, 39 relevant publications were selected, seven of which were used in our review. To access the quality of articles, we used the Cochrane checklist. The collected articles were scored independently by three authors, and the publications with the eligibility criteria were included in our review. No article was excluded due to quality assessment. *Results and Conclusion*. Our findings indicated that GM products had no adverse effects on infertility indices such as the sperm head, sperm motility, sperm abnormality, and fertility indices. Long-term research still seems to be strongly necessary to ensure that the use of GM plants does not cause any harm to consumers, especially in infertility area.

## 1. Introduction

Genetically modified (GM) plants have been generated in agriculture since 1993. In most cases, the objective is to introduce a new characteristic plant that is not naturally present in the species. DNA has been modified by genetic engineering techniques in GM to ameliorate the desired characteristics such as resistance to pesticides or improved nutritional content. Over the past 30 years, transgenic cereals have been rapidly developing. In 1996, the global planting areas of GM plants were 1.7 million hectares while in 2012, they were 170 million hectares and are still rising by 6% in 2012. GM cereal crop varieties are categorized into two generations. Initially, their use was due to the resistance of insects and the reduction of herbicides and pesticide usage, and in the second generation, the use of these products was aimed at producing them more nutritious. The most typical GM crops are soybean, maize, rice, and colza. With the increase in the growth of GM crops, concerns as to the adverse effects of GM crops have risen in the community. In other words, GM products potentially could have both positive and negative impacts on health. Moreover, GM products' food safety is still a controversial issue, and the public does not completely accept transgenic products [1].

GM foods are considered to be responsible for the development of infertility which appears to be a major global issue. It is revealed that 8% to 12% of reproductive-age couples worldwide will be affected, and approximately 40–50% of all infertility cases are due to “human factors” [2, 3]. Infertility refers to no pregnancy following a year of regular unprotected sex. As a multifactorial disease, infertility can be caused by many medical and nonmedical conditions. Since the mid-1990s, GM products have gradually joined the agricultural supply of food, raising concerns as to their possible adverse health effects, including infertility. In addition, a meta-analysis which reviewed articles between 1973 and 2011 reported an average sperm count reduction of 50–60% [4]. With the emergence of the potential for GM plant production, these plants run a higher infertility risk. It is probable that GM crops affect the sperm parameters such as morphology, motility, or abnormal steroid hormones, possibly influencing the infertility index. It is necessary to conduct research in this area to meet the demands of the community and tailor an appropriate message.

Despite the increase in the use of GM crops, the benefits and risks associated with GM products remain uncertain, especially the potential fertility threats over the recent years. The present review seeks to assess the GM plants' potential impacts on sperm parameters, including sperm head, sperm motility, sperm abnormality, and fertility index through a systemic review and meta-analysis.

## 2. Methods

### 2.1. Search Strategy

A structured literature search was independently performed by three authors (P.K, MR.H, and MH.SH.) in Scopus, Web of Science, PubMed, and Embase in January 2019. Search strategy was considered as follows: ((“Infertility” OR “Male Infertility” OR “Female Infertility” OR “Infertile^*∗*^” OR “Sterility” OR “Reproductive Sterility” OR “Subfertility” OR “Sub-Fertility”) AND (“Genetically Modified Food” OR “Genetically Modified Plant” OR “Genetically Modified crop” OR “Genetically engineered crop” OR “Genetic manipulated crop” OR “Transgenic crop” OR “transgenic food” OR “genetically altered food” OR “genetically altered crop” OR “genetically altered plant” OR “Bioengineered food” OR “Bioengineered crop” OR “Bioengineered plant” OR “Genetically Modified Organisms”)).

### 2.2. Inclusion and Exclusion Criteria

**Figure 1:**
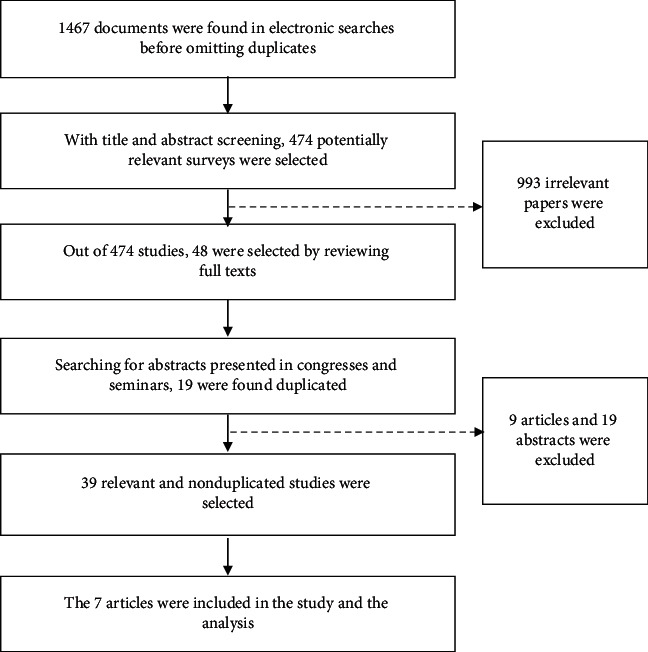
Follow diagram of systematic review and searches for effect of genetically modified food on infertility indices.

A total of 1467 publications were obtained by the search strategy after eliminating the duplicates. Moreover, the review only included articles written in English language. Other pertinent peer-reviewed publications were chosen (“snowballing”) from the references lists in the selected publications. To assess the GM crops' effects on infertility, experimental studies designed with the control group were selected. On the basis of abstract screening and full-text reviewing, 39 relevant publications were selected, seven of which were used in our review (Figure 1). To access the quality of articles, STROBE checklist was used. The collected articles were scored independently by three authors (P. K, H. J., and MR. H.), and the publications with the eligibility criteria were included in our review. No articles were excluded due to quality assessment.

## 3. Results

Three studies reported sperm parameters over 90 days, while others indicated shorter periods [5–7]. One study was conducted on mice [8] and rats [5–7, 9–11]. GM crops were rice in 4 studies [6–9] and maize [5] and potato [11] in the other studies. Infertility was evaluated based on different indices such as sperm motility, head count, morphology, fertility index, gestation length, live-birth rate, mating index, and gender ratio The characteristics of the studies are declared in Table 1.

Sperm shape abnormality was assessed in five studies [5–9], while sperm head count and motility were reported in 4 studies [5–7, 9]. No significant differences were detected in these items The results are explained in Table 2.

### 3.1. Sperm Parameters

Sishuo Cao et al. showed that GM groups and its negative control group did not significantly differ in terms of the frequencies of sperm shape abnormality at high, middle, and low doses of Cry1C protein groups. Sperm abnormalities were significantly higher in the positive control group compared with the negative control group [8]. According to both Guo et al. and Wang et al.'s studies, the results of sperm parameter test (including sperm motility, sperm head counts, and epididymis sperm morphology) were not significantly different (*p* > 0.05) between the three intervention groups (diets containing GM, near isogenic line, and standard diet) after the 90-day feeding trial [5, 6]. In the study implemented by Rhee et al., the sperm motility ranged from 80 to 100% in all groups. There were no significant differences in the percent of motile sperms. Nonmotile sperms showed 0% [11].

### 3.2. Testicular Marker Enzymes

The toxic effects of GM on the activity of testicular marker enzymes were assessed, and the results showed that the activity of testicular function enzyme acid phosphatase (ACP), lactic dehydrogenase (LDH), and succinate dehydrogenase (SDH) had no significant differences with standard diet and the control group (*p* > 0.05) [9]. In a study by Zhou et al. [7], no negative impacts were observed on the reproductive ability of the three generations (F0–F2) of rat parents provided with transgenic rice over the periods of mating and gestation in terms of fertility index, copulation index, live-birth rate, and gestation length. There is no evidence as to the impact of GM products on the female rats' estrous cycle. Regarding development, number of pups, birth, and the gender ratio of pups in F1–F3 generation offspring, no significant differences were seen between the control or standard diet group and the GM rice group.

Nor were any significant differences observed as to the male rats' reproductive indices including the sperm count and morphologically abnormal sperms; that is, GM rice did not adversely impact the reproductive system of the male rats. As to the male rats on the GM rice diet, the testis cell cycle's tetraploid and diploid population were lower (*p* < 0.05) compared with those on the standard diet, not the non-GM isogenic diet [7]. Testicular cells are capable of secreting androgen, testosterone in particular (95%); the testicular cells' secretion function was influenced by toxic effects. However, in terms of serum androgen levels, no differences were seen, and regarding the testes, no macroscopic or histological negative impacts were observed. Live-birth rate, gender ratio of pups, and gestation length were not significantly different between the groups during all generations (F0–F2) [7].

### 3.3. Mating and Fertility Indices

Rhee et al. reported that the fertility and mating indices of the potato-treated groups, similar to all the other groups, varied from 85 to 100%. Regarding F0 production, on the other hand, GM potato-treated male (72.0%) and female (78.3%) groups had lower fertility indices in comparison with the control and non-GM groups. The GM diet did not change the patterns of estrous cycle. In all groups, the length range of gestation was 21-22 d. Similar to the control and non-GM groups, the mean litter size (*n*) in the GM potato-treated group varied between 10 and 13. In all groups, the range of sperm motility was 80–100%, and no significant differences were observed regarding motile sperm percentage. Nonmotile sperms revealed 0%. Gestation length and delivery index (%) were not significantly different between the groups [11].

Tyshko et al. reported no GM maize influence on the fertility of animals, which is similar to the aforementioned studies: in both groups, the efficiency of mating was observed to fall under the normal expected range in the provided experimental conditions (71–92%, 79–80%, and 77–87% in the Fb, F0, and F1 generations). The percentage of nonfertile males ranged from 0 to 13%. Moreover, the two groups were not different as to progeny prenatal development in F0–F2 generations. No abnormalities were found in the physical progress associated with the weight and length of F0–F2 progeny or pups. In the control and test groups, the average number of pups per litter was in the expected range (9.53–11.80). The groups were not statistically different, and the male/female ratio slightly differed concerning each generation. Nevertheless, these variations had similar trends and fell under the normal ranges expected for Wistar rats. Accordingly, these findings should be deemed as the direct evidence of the absence of reproductive toxicity [10].

## 4. Discussion

Despite the controversies regarding GM products and health, concerns are now being raised about the potential negative effect on human fertility. There are several studies and reviews of the impact of GM products on human health, but little information is available on GM consumption and infertility. The results of our systemic review, including seven experimental studies, showed that the use of GM products in experimental studies did not represent significant differences in sperm parameters such as sperm abnormality, sperm motility, sperm head, and fertility indices.

There are several scientific reports and recommendations considering the adverse health effects of GM products. European countries have banned the use of GM foods in Europe [12, 13]. In addition, the American Academy of Environmental Medicine (AAEM) released its policy paper on GM foods on 8 May 2009, which included research on several peer-reviewed studies. They state clear policies as follows: “there is more than a casual association between GM foods and adverse health effects. There is causation as defined by Hill's Criteria in the areas of strength of association, consistency, and specificity, biological radiant, and biological plausibility.” However, the US government and GM manufacturers claimed that the consumption of GM products is healthy [14]. In addition, many farmers agree with GM production [15]. Based on the aforementioned points, it appears that the financial aspect of GM products might contribute to these distinct strategies. Also, Jeffrey Smith's report showed that avoiding GM consumption can improve health, including digestion: 85.2%, exhaustion, low energy: 60.4%, overweight and obesity: 54.6%, food allergies or sensitivities: 50.2%, anxiety or depression: 51.1 %, joint pain: 47.5%, and hormonal problems: 30.4%. In this analysis, however, the author did not report the relationship between avoiding GM consumption and the fertility indices [14].

There are several potential mechanisms for the impact of GM products on sperm parameters. Since we reviewed the studies of feeding with rice or maize, no significant sperm parameter abnormality was observed; however, the lines of evidence are conflicting in the potato feeding group, which could be due to potato's antioxidant and radical scavenger activity. Some studies have demonstrated that foods with antioxidant activity have positive effects on fertility factors. [16, 17]. Based on such literature, the antioxidant activity of food products can affect the quality and quantity of the sperms in males; thus, it is assumed that the antioxidant activity of transgenic products may have been less than that of nontransgenic products. In 2011, Xu et al. have suggested that certain oxidoreductase activity indices such as superoxide reductase, polyphenol oxidase, peroxidase, and catalase activity were significantly lower in some GM than non-GM products [18].

On the other hand, a study in 2007 conducted by Kodrík et al. demonstrated that GM potato could increase some oxidative stress related hormones such as adipokinetic hormones (AKHs) in *Leptinotarsa decemlineata* insect fed with GM potato [19]. The above evidence may be a reason for the reduction of oxidative stress-reducing substances in some foods, especially potatoes, and this may confirm the influence of the type of food on the design of the study. As to the other corns, further analysis and studies are needed.

The result showed that there were not any differences in the animals for testing GM products. Although no specific animal has been suggested to investigate the effects of transgenic products, laboratory rodents have assumed to be more appropriate in some research [20].

Review of the literature shows that the studies performed in just one generation have not seen much change, but those on more than two generations have reported some changes. Therefore, researchers who intend to study in this field should design their study so that more than two generations can be studied and there may be complications in subsequent generations.

With a few exceptions to the results of the published studies over the last decade, GM products could be safe and beneficial [21, 22]. Although no significant changes were reported in this review study, the slight variations indicated that these products might have longer-term effects. Also, we can consider whether the use of toxins and their effects or the use of transgenic foods can harm the people's health [1]. Also, support for GM production could reduce the use of pesticides by farmers. Many of the pesticides were associated with health and environmental issues [23]. Evidence has shown that there is no support to suggest GM production as harmless to human health. Although our finding of the experimental studies did not show a significant adverse health effect, the results cannot be generalized to humans and they need to be interpreted with caution.

To promote the worldwide health, safety assessment of GM is emphasized in national and international standard. Therefore, the need for independent research on the health effects of GM food is now urgent [24]. One approach of the safety assessment is to compare the novel protein or new species of crops to known food allergens or protein toxicity. For example, the Cry1C amino acid sequence was compared to protein databases of known allergens and toxins of the Cry1C protein. Another approach should focus on the assessment of unintended effects that could result from gene insertion in GM products. We strongly suggest that the safety of GM foods should be assessed on a case-by-case basis in a comprehensive health perspective. In addition, improving the principles for the human health risk analysis of GM foods in the Codex Alimentarius Commission (Codex) is helpful.

While the government policy has embraced GM technology and GM food, there has been public concern about the benefits of GM products around the world. Our findings indicated that GM products had no adverse effects on infertility indices such as sperm head, sperm motility, sperm abnormality, and fertility indices. Long-term research still seems to be strongly necessary to ensure that the use of GM plants does not cause any harm to consumers, especially in infertility area. To promote public attitudes, future research should be conducted to determine whether long-term effects of GM plant intake can affect nutritional epigenetic, mutagenicity, teratogenicity, and carcinogenicity.

## Figures and Tables

**Table 1:** Characteristics of the studies on the effects of genetically modified crops on animal's fertility.

No.	Author	Year	Sample size	Animal	Generation	Duration	Product	% of GM crops	Outcome
1	Gyu Seek Rhee	2005	25 in each group and in each generation (total: male 75, female 75)	Sprague Dawley rats	5	10 weeks	Potato	5%	Fertility index, mating index, gestation index (%), gestation length, delivery index (%), litter size, estrous cycle
2	Sishuo Cao	2010	12 mice for each group (6 males and 6 females per treatment)	Mice	1	5 days	Rice: PET-30a(+)-Cry1C-rcp-BL21 (DE3)	5 g (Cry1C protein)/kg	Sperm abnormality
3	Wang	2013	24	Wistar rats	1	90 days	Rice TT51	60%	Sperm motility, sperm morphology, sperm head counts
4	Zhou	2014	60	Sprague Dawley rats	3	13 weeks	Rice	70%	Sperm morphology, sperm head counts, copulation index (%), fertility index (%), gestation length (days), live-birth rate, no. of pups, birth, gender ratio of pups, estrous cycle (days), sperm parameters
5	Tyshko	2014	380 adult animals and 1540 pups	Wistar rats	3	90 days	Maize	32–33%	Fertility index, number of alive fetuses, total number of pups, mean litter size, ratio ♂/♀ in the litter
6	Guo	2015	30	Male Wistar rats	1	90 days, 13 weeks	Maize BT799	84.7%,	Sperm motility, sperm morphology, sperm head counts
7	Wang Er Hui	2016	15 males/30 females, each group; 8 male offspring rats	Wistar rats	2	70 days	Rice TT51	60%	Sperm motility, sperm morphology, sperm head counts, testicular function enzyme ACP, LDH, and SDH activity

ACP, acid phosphatase; GM, genetically modified; LDH, lactic dehydrogenase; SDH, succinate dehydrogenase.

**Table 2:** Effects of the genetically modified crops on animal's fertility (based on sperm head count, sperm motility, sperm abnormality, and fertility index).

No.	Author	Year	Sperm head (1000000/Ml)			Sperm mortality%			Sperm abnormality%			Fertility index^a^			Clinical pathological parameter
			GM	Non-GM	Standard	GM	Non-GM	Standard	GM	Non-GM	Standard	GM	Non-GM	Standard	
1	Gyu Seek Rhee	2005	—	—	—	80–100%	80 to 100%	80 to 100%	—	—	—	72–100%^*∗*^	88–100%	92–100%	No adverse effects on the multigeneration reproductive-developmental ability. Nonmotile sperms showed 0%.

2	Sishuo Cao	2010	—	—	—	—	—	—	Cry1C.DOSE.1250 = . 1.50 ± 0.5. Cry1C.DOSE.625 = 1.24 ± 0.6. Cry1C.DOSE.125 = 1.38 ± 0.4	Positive control = 4.86 ± 1.2^*∗*^	Negative control = 1.28 ± 0.5	—	—	—	<16 sperm abnormalities per 1000 cells No adverse effect

3	Wang	2013	160.59 ± 34.91	164.17 ± 19.33	157.45 ± 24.51	82.94 ± 10.28	85.56 ± 8.23	86.37 ± 8.57	9.13 ± 1.90	10.25 ± 2.38	8.88 ± 1.36	—	—	—	No significant differences were detected in terms of sperm motility; sperm head counts and morphology of epididymis sperm between groups

4	Zhou	2014	5.66 ± 1.83	5.51 ± 1.74	5.48 ± 1.49	—	—	—	1.52 ± 0.34	1.53 ± 0.28	1.60 ± 0.21	85%	85–90%	85–90%	Some statistically significant differences were observed in rats consuming the high amylose rice, differences were generally of small magnitude, herefore not considered to be biologically meaningful or treatment related.

5	Tyshko	2014	—	—	—	—	—	—	—	—	—	71–80%	79–92%	—	Lack of any reproductive toxicity

6	Guo	2015	32.35 ± 1.79	30.50 ± 2.62	31.69 ± 2.33	87.84 ± 4.00	83.91 ± 6.02	84.06 ± 5.90	9.44 ± 0.98	9.00 ± 0.93	9.00 ± 0.69	—	—	—	No significant differences in sperm parameters with the diets containing transgenic BT799, Zhen58 and the control, no treatment-related side effects on the reproductive system of male rats.

7	Wang Er Hui	2016	185.49 ± 20.35	173.81 ± 16.49	185.15 ± 27.89	86.02 ± 8.72	83.10 ± 7.05	85.29 ± 10.27	6.38 ± 2.11	6.63 ± 1.93	6.25 ± 2.11	—	—	—	No significant differences on the reproductive system of male offspring rats compared with MingHui63

GM, genetically modified. ^*∗*^Significant from control (*p* < 0.05) in F0 group but not in F1, F2, F3, and F4. ^a^Fertility index (%) = (no. of females pregnant/no. paired) × 100 (ranges from different generations).

## Additional Points

*Knowledge Translate* (KT). What is already known in this topic: GM foods are considered to be responsible for the development of infertility which appears to be a major global issue. Risks associated with GM products and the potential fertility threats over the recent years remain uncertain*. The Addition of This Article.* Based on the current research, GM products had no adverse effects on infertility indices such as sperm head, sperm motility, sperm abnormality, and fertility indices. Long-term research still seems to be strongly necessary.
